# Interactive Data Visualization for HIV Cohorts: Leveraging Data Exchange Standards to Share and Reuse Research Tools

**DOI:** 10.1371/journal.pone.0151201

**Published:** 2016-03-10

**Authors:** Meridith Blevins, Firas H. Wehbe, Peter F. Rebeiro, Yanink Caro-Vega, Catherine C. McGowan, Bryan E. Shepherd, The Caribbean, Central, South America Network for HIV Epidemiology (CCASAnet)

**Affiliations:** 1 Vanderbilt University School of Medicine, Nashville, Tennessee, United States of America; 2 Department of Infectious Diseases, Instituto Nacional de Ciencias Médicas y Nutrición Salvador Zubirán, Mexico City, Mexico; University of Pennsylvania School of Medicine, UNITED STATES

## Abstract

**Objective:**

To develop and disseminate tools for interactive visualization of HIV cohort data.

**Design and Methods:**

If a picture is worth a thousand words, then an interactive video, composed of a long string of pictures, can produce an even richer presentation of HIV population dynamics. We developed an HIV cohort data visualization tool using open-source software (R statistical language). The tool requires that the data structure conform to the HIV Cohort Data Exchange Protocol (HICDEP), and our implementation utilized Caribbean, Central and South America network (CCASAnet) data.

**Results:**

This tool currently presents patient-level data in three classes of plots: (1) Longitudinal plots showing changes in measurements viewed alongside event probability curves allowing for simultaneous inspection of outcomes by relevant patient classes. (2) Bubble plots showing changes in indicators over time allowing for observation of group level dynamics. (3) Heat maps of levels of indicators changing over time allowing for observation of spatial-temporal dynamics. Examples of each class of plot are given using CCASAnet data investigating trends in CD4 count and AIDS at antiretroviral therapy (ART) initiation, CD4 trajectories after ART initiation, and mortality.

**Conclusions:**

We invite researchers interested in this data visualization effort to use these tools and to suggest new classes of data visualization. We aim to contribute additional shareable tools in the spirit of open scientific collaboration and hope that these tools further the participation in open data standards like HICDEP by the HIV research community.

## Introduction

In the practice of epidemiology, data visualization has been of great importance historically [1], whether for exploration of data structures preparatory to analysis [2], for interpreting patterns of events in populations over space and time [3], or for more clearly communicating inferences drawn from completed analyses [4]. Data visualization has also been important in our understanding of the HIV epidemic [5, 6]. Data animations can improve figures by allowing the display of a temporal dimension [3]. In many static plots, the requisite data dimensions consume all the display space precluding the opportunity to add the temporal dimension without compromising the clarity and effectiveness of conveyed information. Static snapshots taken of plots at regular time intervals can be strung together to form frames in a video animation, the direction and speed of which can be altered by the user. For example, recent work elucidated the CD4 and viral load response to antiretroviral therapy using a dynamic visual display [7].

While various data visualization techniques in related domains, including geographic information systems [8, 9], social networks [10], and bioinformatics [11] have been proposed and analyzed, they mostly require loading the data into tool-specific stores and formatting that data according to ad hoc syntax. A recent systematic review of data visualization tools for infectious diseases suggested that future developers focus on the broader contexts of available data, team collaboration, and interdisciplinary needs [12]. Existing tools attract users when they are free, interactive, transparent, and have a limited learning curve. We maintain that an open standard unifying syntactic and semantic definitions coupled with an open set of data analytic and visualization tools would provide sufficient incentive for the community to incrementally build and enhance such tools [12–16].

We describe a tool built with open access software and data exchange standards to promote visualization of HIV cohort data. We identified classes of regularly used plots for which an additional temporal dimension—displayed through interactive animation—can increase their appeal and explanatory power. We demonstrate the tool using HIV cohort data from the Caribbean, Central and South America network for HIV research (CCASAnet).

## Methods

### Cohort Description

CCASAnet is a shared repository of HIV cohort data from sites in Argentina, Brazil, Chile, Haiti, Honduras, Mexico and Peru. The collaboration was established in 2006 as part of the International Epidemiologic Databases to Evaluate AIDS (IeDEA; www.iedea.org) with the purpose of collecting retrospective clinical HIV data to describe the unique characteristics of the epidemic in the region [17]. The Vanderbilt University Medical Center Institutional Review Board approved this project. Local centers de-identified all data before transmitting it to the CCASAnet Data Coordinating Center at Vanderbilt University, so no informed consent was required.

### Data Exchange Standard

Cross cohort collaborations have long been hindered by utilizing different protocols for data exchange. In an effort to reduce the workload of data extraction and speed up the time to analysis, an HIV Cohort Data Exchange Protocol (HICDEP, available at http://www.hicdep.org/) was developed and widely disseminated in 2004 [18]. In 2010, CCASAnet adopted a data transfer protocol based on HICDEP to support and streamline data harmonization between the multiple sites. The CCASAnet Data Coordinating Center has leveraged this open standard to build a suite of data visualization tools that can be shared with the HIV cohort community and beyond as open source tools. While the results in this paper use actual CCASAnet data, example datasets have been made available to readers in order to practice using the tools highlighted in this paper (http://biostat.mc.vanderbilt.edu/ArchivedAnalyses).

### Data Visualization

Currently, there are three classes of plots requiring patient-level or country-level data (described below). The graphics are implemented using R statistical language and encoded using MEncoder. The R code may be downloaded from our GitHub repository (https://github.com/CCASANET/dataviz), applied to HICDEP compliant data, and customized as indicated in the written instructions or with the aide of an instructional video (http://biostat.mc.vanderbilt.edu/ccasanet/dataviz/instructions.htm). Current classes of plots are the following:

1. Longitudinal plots / event probability curves. This panel of graphics was motivated by common figures used to describe HIV therapy outcomes, including spaghetti plots, density curves, and Kaplan-Meier plots [19–21]. Longitudinal measures (e.g., CD4 count) with smoothed curves are viewed alongside event probability curves allowing for simultaneous inspection of outcomes (e.g., mortality) stratified by patient classes (e.g., AIDS status at ART initiation). The smoothed LOESS curves are fit once over the whole time span using locally-weighted polynomial regression [22]. Density curves are shown in the margins demonstrating changes of the longitudinal measure as its trajectory grows in the frame; these changes in the density cannot be effectively visualized in a single static frame. Inputs per subject include: a longitudinal continuous measure and dates (e.g. CD4 count), an event indicator and corresponding date (e.g. death), a start date (e.g. combination antiretroviral therapy [cART] initiation), and a classifier (e.g. AIDS).
2. Bubble plots. Inspired by Hans Rosling’s popular TED talks on world population statistics and his Gapminder project [23], this graphic shows changes in indicators over time allowing for observation of group level dynamics. Bubble plots show three dimensions of data including the placement on each of two axes and the size of the bubble, and a fourth dimension is added by showing the change over time in video presentation. This graphic takes as input: two indicators (e.g. CD4+ count < 200 cells/μL or AIDS diagnosis), one date (e.g. enrollment into HIV care), and one classifier (e.g. study site).
3. Heat maps. World maps are commonly used to show the burden of global HIV disease [24, 25]. By displaying these maps over time, we can simultaneously view the spatial element of cohort data along with the population trends. A heat map shows borders of countries filled in with darker colors for high proportions and lighter colors for low proportions. This graphic takes as input a dataset with one indicator record for each country and year. There is a sample R script (cd4_base_country.R) that demonstrates how a user might generate this country-level dataset using patient-level data.

The three classes of plot were tested independently from the developer (MB) by two CCASAnet members (YCV and MJG). The step-by-step instructions for users include:

1. Download ZIP files from the GitHub repository: https://github.com/CCASANET/dataviz
2. Unzip the downloaded files to project location
3. Copy HICDEP compliant HIV cohort data to the input folder
4. Download and install R
5. Download and install RStudio
6. Edit the input/panel1_specs.csv or input/panel1_specs.csv or input/map1_specs.csv specifications to fit the project needs
7. Open code/panel1_graphic.R or code/panel2_graphic.R or code/map1.R code using R Studio
8. Change the working directory to the project location (i.e. the directory containing code, input, output), and source the file.
9. Results are viewable in output/*_viewer.html.
10. The user may optionally compile the graphics written to output/scroll_images/panel_*.png as a video using MEncoder or other encoding programs.

Users may also view step-by-step video instructions at our website (http://biostat.mc.vanderbilt.edu/ccasanet/dataviz/instructions.htm).

## Results

**Fig 1:**
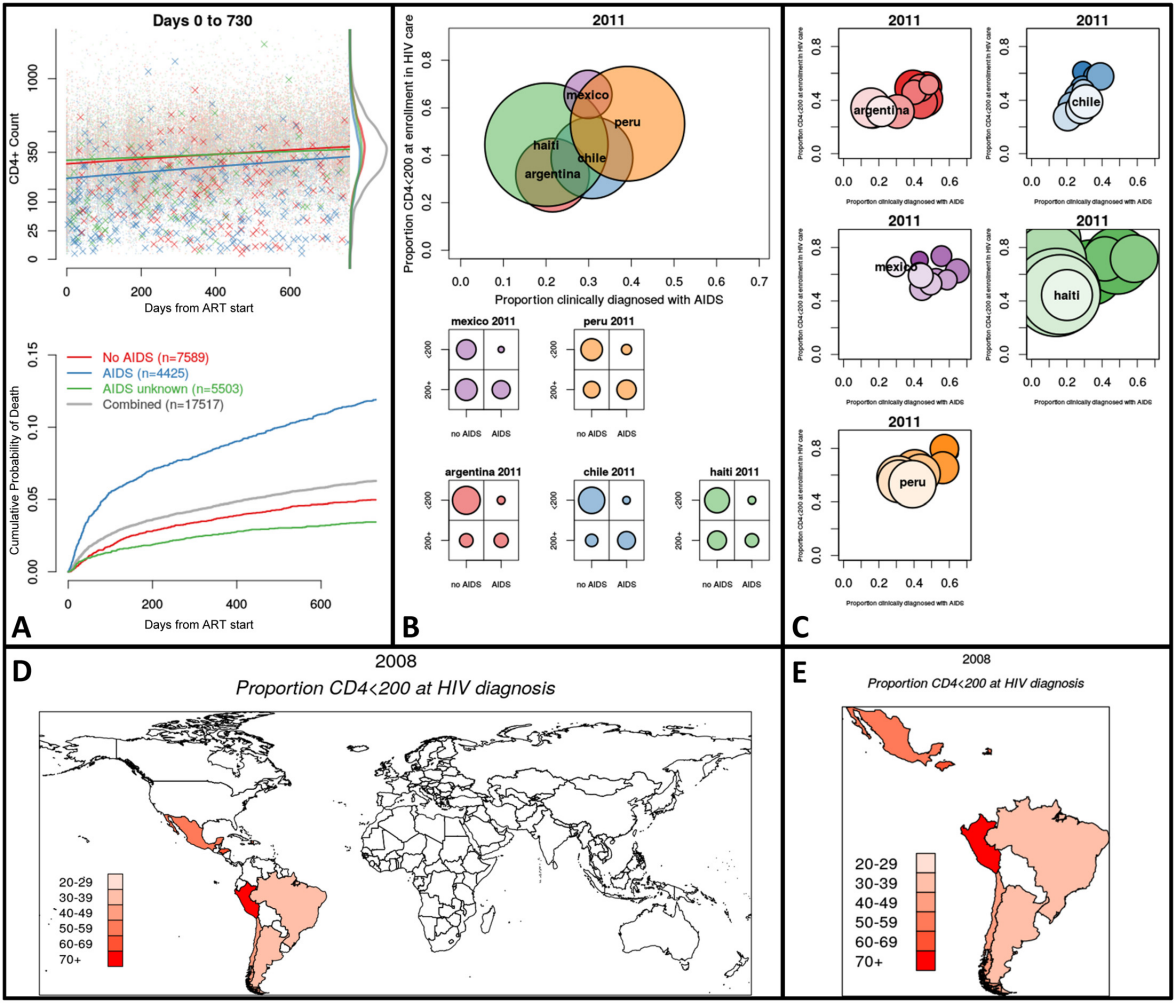
Featured frames from animated graphics (see http://biostat.mc.vanderbilt.edu/ccasanet/dataviz/examples.htm). *Fig 1A*, *Example Panel 1*: Immunologic recovery and mortality two years following cART initiation, stratified by clinical stage at ART initiation. Top panel: Time since ART initiation by CD4+ count. The dots mark observations and the Xs mark deceased patients at time of death and their last CD4+ count. Density curves show the two-year CD4+ count distribution by AIDS status. Bottom panel: Kaplan-Meier curves showing cumulative probability of death separated by AIDS status. *Fig 1B*, *Example Panel 2*: Distribution of low CD4+ count and AIDS diagnosis at enrollment by region in 2011. Top panel: Bubble plot showing proportion of patients enrolled during 2011 who are clinical AIDS by the proportion with low CD4+ count; bubbles are proportional to the number of patients enrolled in 2011. Bottom panel: Marginal distributions of clinical AIDS by low CD4+ count for each country in 2011. *Fig 1C*, *Example Panel 3*: Distribution of low CD4+ count and AIDS diagnosis at enrollment by region in 2011. Bubble plot showing proportion of patients enrolled during 2011 who are clinical AIDS by the proportion with low CD4+ count. The top and lightest colored bubble is the current year (2011). The bubbles beneath represent prior years and darken as time passes. *Fig 1D*, *Example Map 1*: World heat map showing proportion of newly diagnosed patients with low CD4 count in 2008. Countries with lightest shade of red have 20–29% of patients with low CD4+ count at HIV diagnosis. The CShapes dataset by Weidmann and Gleditsch is licensed under a Creative Commons Attribution-NonCommercial-ShareAlike 4.0 International License [26]. *Fig 1E*, *Example Map 2*: Country heat map showing proportion of newly diagnosed patients with low CD4 count in 2008. Countries with lightest shade of red have 20–29% of patients with low CD4+ count at HIV diagnosis. The CShapes dataset by Weidmann and Gleditsch is licensed under a Creative Commons Attribution-NonCommercial-ShareAlike 4.0 International License [26].

Examples of the output of three classes of plots based on CCASAnet data are summarized below; animations are best visualized at our website (http://biostat.mc.vanderbilt.edu/ccasanet/dataviz/examples.htm) and frames from the example animations are provided in Fig 1. All plots come with example user specifications as outlined in Table 1; users may directly edit the specifications in a CSV document to change the various inputs and parameters for the plots as detailed below.

**Table 1:** User modifiable specifications read by R to create interactive graphics referred to as Panel 1, Panels 2–3, and Maps 1–2.

*Panel 1*: *Animated scatter and Kaplan-Meier plots*, *as shown in* Fig 1A		
**name**	**specification**	**details**
id	patient	Unique identifier in datasets
longtablename	lab_cd4	Table name (same as CSV name during data load)
longvar	cd4_v	Longitudinal variable name (must be present in table above)
longvardate	cd4_d	Longitudinal variable date name (must be present in table above)
longsubset	function(longvar) longvar > 0 &! is.na(longvar)	Function to be applied to subset data in longtablename (will currently be applied only to longvar; eg, function(x) x > 0) [default is no missing]
eventtablename	follow	Table name (same as CSV name during data load)
event	death_y	Event variable name (must be present in table above)
enddate	l_alive_d	End date name (must be present in table above)
grouptablename	basic	Table name (same as CSV name during data load)
group	aids_y	Grouping variable name (must be present in table above)
groupsubset		Set of allowable values for groups [default is all unique groupings]
starttablename	art	Table name (same as CSV name during data load)
startdate	art_sd	Start date name (must be present in table above)
starttype	first	Specification for identifying one start date if there are multiple per unique ID (options are "first" or "last") [default is "first"]
longvartrans	sqrt	Transformation for longvar (currently allow for "sqrt", "log", "log10") [default is no transformation using the identity function "I"]
maxtime	730	Numeric maximum time in days for follow-up (start to end date) [default is 730 days]; this means that the plot will run from 0 to maxtime days.
long2eventwindow	360	Numeric allowable time to pass between longvar collection and event date in order for value to be attributed to event. [default is 360 days]
longvarlim	c(0,1500)	Two numeric values for limit of longvar (y-axis pane 1) [default is .5th and 99.5th quantiles]
problim		Two numeric values for limit of event rate (y-axis pane 2) [default is 0 and 1.4 times highest group rate]
longticks	c(0,25,100,150,350,500,1000,2000,5000)	Any length of numeric values to put tick marks and labels on longvar y-axis (original scale) [default is 5 points using pretty function]
longlabel	CD4+ Count	Label for y-axis of scatterplot [default is "Longitudinal Value"]
timelabel	Days from cART Start	Label for y-axis of scatterplot [default is "Days"]
eventlabel	Cumulative Probability of Death	Label for y-axis of Kaplan-Meier plot [default is "Probability of Death"]
grouplabels	0 = No AIDS|1 = AIDS|9 = AIDS unknown	Group label for plot legend. Must be a unique value of group variable, equal sign, label separated by pipes. This is useful if the values of group are themselves not informative. [default is "Group" followed by unique value]
*Panels 2–3*: *Animated bubble and marginal plots*, *as shown in* Fig 1B and 1C		
**name**	**specification**	**details**
vartable	basic_cd4	table name (same as CSV name during data load)
var1	basic_cd4$aids_cl_y = = 1	R code that creates an indicator variable using variables in vartable. Or use an indicator already appended to the existing dataset.
var2	basic_cd4$cd4_v_cmp < 200	R code that creates an indicator variable using variables in vartable. Or use an indicator already appended to the existing dataset.
vartablesubset	format(convertdate(baseline_d,basic_cd4),"%Y") > 1998 & format(convertdate(baseline_d,basic_cd4),"%Y") < 2016 & basic_cd4$aids_cl_y %in% c(0,1) &! is.na(basic_cd4$aids_cl_y)	R code that creates an indicator variable that will be used to subset the original vartable using variables in vartable
eventdate	baseline_d	Date corresponding to relevant data collection (e.g., date of enrollment).
eventperiod	year	How to discretize time for the different frames of the bubbleplot. [default is year, allowable values are month, quarter, year, and missing]
group	site	Grouping variable in vartable for the different bubbles.
var1label1	AIDS	Label of group for which var1 is 1 or TRUE.
var1label0	no AIDS	Label of group for which var1 is 0 or FALSE.
var2label1	<200	Label of group for which var2 is 1 or TRUE.
var2label0	200+	Label of group for which var2 is 0 or FALSE.
var1label	Proportion with clinical AIDS	Axis label for var1.
var2label	Proportion CD4<200 at enrollment in HIV care	Axis label for var2.
minnum	10	Set minimum number of observed units for a bubble to be drawn [default is 10].
*Maps 1–2*: *Animated world and region heat maps*, *as shown in* Fig 1D and 1E		
**name**	**specification**	**details**
countrytable	basic_cd4_country	Table with one row per country and time point—example manipulation of existing DES tables in code/cd4_base_country.R
var	var1_prop	Value of attribute to be mapped in Proportion (range: 0–1) or Percentage (range: 0–100)
country	country	Variable with ISO-3 country code
year	year	Time point for temporal connection of maps
varlabel	Proportion CD4<200 at HIV diagnosis	Label for attribute

### Panel 1: Immunologic recovery and mortality following cART initiation, stratified by clinical stage at ART initiation

Fig 1A shows the final frame of these longitudinal plots / event probability curves applied to 17,517 patients. Each frame corresponds to a 1-day increment from date of cART initiation. The top panel is a scatterplot of days on cART by CD4; observed values are marked with a semi-transparent dot at the day of observation and an X at the day of death (the last observed CD4 count is used as long as it was recorded within 12 months of death). Density curves show the most recent distribution of CD4. The bottom panel shows Kaplan-Meier estimates of the probability of death. In the CCASAnet cohort, patients initiating cART immediately separate into two groups, higher CD4 and lower CD4 with AIDS status at initiation, and patients initiating cART with AIDS have increased probability of death. It is interesting to also note that conditional on survival past one year, the CD4+ counts for these groups become similar. This data visualization may be useful to look at sex or age-group differences in HIV care and treatment outcomes.

### Panels 2–3: Distribution of low CD4 count and AIDS diagnosis at enrollment by region during 2000–2014

In our example (Fig 1B and 1C), each frame corresponds to calendar year of enrollment into HIV care. In Fig 1B, the top panel is a bubble plot with bubbles representing regions, coordinate locations corresponding to observed proportions for each indicator, and bubble size proportionate to the number of newly enrolled individuals. The bottom panel includes contingency plots that show marginal allocations of both indicators within the classifier. In Fig 1C, each region has a panel showing a trail of bubbles as time progresses, with coordinate locations corresponding to observed proportions for each indicator, and bubble size proportionate to the number of newly enrolled individuals. From the plots we can see that in most sites the proportions of new enrollees with low CD4 count (<200 cells/*μ*L) and clinical AIDS has decreased over time. From the bottom panel of Fig 1B, we observe that the marginal proportion of patients with an AIDS diagnosis and low CD4+ count represent a non-zero share of the country-level enrollment population in 2011; this counter-intuitive result reveals the situation where subjective and objective measures do not always agree in the data. Informed by this visualization, a CCASAnet researcher may next want to formally test whether patients seem to be entering HIV care and treatment in earlier clinical stages as the epidemic response increases or as the program matures. Both panel graphics are created by the same set of input specifications and R code. For this example, it is necessary to calculate baseline CD4 count and merge with existing data; the creation of this dataset may be optionally aided using the example code in add_cd4_base.R. If derived variables such as baseline CD4 count are required, the user may employ our optional example code or instead add this derived variable using familiar data manipulation software.

### Maps 1–2: Country heat maps showing proportion of newly diagnosed patients with low CD4 count during 2000–2014

In our example (Fig 1D and 1E), the proportion plotted corresponds to patients diagnosed with CD4+ cell count < 200 cells/μL. The first map (Fig 1D) shows the entire world to give context to the countries in the cohort, and a second map (Fig 1E) is produced that highlights only those countries with data in any of the time periods. Both maps are created by the same set of input specifications and R code. It is necessary to input a dataset that has one record for country and time period; the creation of this dataset may be aided using the example code in cd4_base_country.R.

## Discussion

Our goal is to enable HIV researchers to create interactive visualizations of large HIV cohort databases which inspire insight into and even awe at the dynamics of HIV outcomes. Longitudinal plots / survival curves can be used to view changes in CD4+ cell count, HIV viral load, hemoglobin, and other continuously varying measures and the probability of AIDS-defining events, loss to follow-up, death, and other endpoints. Bubble plots can be used to visualize movement in key indicators across relevant groupings over various time periods. Heat maps can be used to provide spatial and temporal context to HIV cohort data. Commonly used graphics in the field of HIV cohort research are made interactive and accessible using open source tools and data exchange standards.

Variations of these visualizations have been incorporated as supplemental figures in CCASAnet manuscripts [27, 28]. Future directions include enhancing the suite of tools with additional classes of data visualization, such as the recently released dynamic visual display of treatment response [7]. While R has an arguably steep learning curve, we have mitigated this through the written and video instructions, hands-on dataset, and by allowing user-modified graphics with simple text file inputs versus editing R scripts. A logical next step in flattening the learning curve of R would be the design of a graphical user interface (GUI) that would allow the user to input the specifications interactively as opposed to editing the specifications in a CSV document. A GUI might also display descriptive statistics to supplement the visualization, or optionally allow for more comprehensive displays of uncertainty such as confidence intervals. Depending on the technical platform, further user interaction with these animations would include the ability to control the direction and speed of the time lapse animation, the ability to highlight and track elements of the plot, and the ability to control parameters that mask or compare alternate scenarios. Researchers interested in this data visualization effort are encouraged to contact the authors and are invited to contribute ideas for additional interactive visualizations that may be openly implemented as part of this research tool set. Building on open standards like HICDEP, we aim to contribute additional shareable tools in the spirit of open scientific collaboration.
